# Genetic Characteristics and Multiple-PCR Development for Capsular Identification of Specific Serotypes of *Campylobacter jejuni*

**DOI:** 10.1371/journal.pone.0165159

**Published:** 2016-10-27

**Authors:** Hao Liang, Aiyu Zhang, Yixin Gu, Yuanhai You, Jianzhong Zhang, Maojun Zhang

**Affiliations:** State Key Laboratory of Infectious Disease Prevention and Control, Collaborative Innovation Center for Diagnosis and Treatment of Infectious Diseases, National Institute for Communicable Disease Control and Prevention, Chinese Center for Disease Control and Prevention, Beijing, China; Massey University, NEW ZEALAND

## Abstract

The polysaccharide capsule (CPS) of *Campylobacter jejuni* is a virulence factor linked to cell surface carbohydrate diversity which mainly determines the serotypes. Thirty-four CPS gene cluster structures have been published and some of them can be distinguished by multiple-PCR. Penner serotypes HS1/44c, HS2, HS4c, HS19, HS23/36c and HS41 are markers for Guillain—Barré syndrome (GBS). The capsules may contribute to GBS susceptibility. Analysis of 18 CPS loci revealed high gene content diversity and a mosaic nature of the capsule loci, which are possibly due to gene gain/loss events, and demonstrated a high degree of conservation of genes within serotypes/serotype complexes. A method of multiple-PCR was developed to distinguish five specific serotypes and three GBS-related serotypes. Primers specific for each capsule type were designed on the basis of paralogs or a unique DNA region of the CPS locus. The multiple-PCR can distinguish the eight serotypes in two PCRs with sensitivity and specificity of 100% using 227 strains of known Penner type. The multiple-PCR method will help to distinguish serotypes simply and rapidly.

## Introduction

*Campylobacter jejuni* is a leading cause of bacterial gastroenteritis worldwide. Guillain—Barré syndrome (GBS) is a serious post-infectious complication (sequela) of *C*. *jejuni* infection. *C*. *jejuni* is similar to Gram-negative mucosal pathogens such as *Haemophilus influenzae* and *Neisseria meningitidis* which can produce a surface capsular polysaccharide (CPS). It has been demonstrated that the CPS gene cluster is located in a hypervariable region of the *C*. *jejuni* genome, and is the major serodeterminant of the Penner or heat-stable (HS) serotyping scheme[1]. Recently, the structures of 34 *C*. *jejuni* CPS gene clusters have been published[2–8], the sizes of which ranged from 15 kb to 34 kb. These clusters contain genes with the function of encoding sugar biosynthesis enzymes, glycosyl transferases and genes of unknown function. Part of these clusters is composed of the conserved heptose biosynthesis genes with the products of *hddC* (putative heptose transferase), *hddA* (putative D-glycero-D-*manno*-heptose 7-phosphate kinase), *gmhA2* (phosphoheptose isomerase) and *dmhA* (GDP-mannose 4,6-dehydratase)[6]. The genes for biosynthesis of O-methyl phosphoramidate (MeOPN), another key structural markers of the capsules of *C*. *jejuni*, were originally identified in *C*. *jejuni* NCTC 11168[9]. MeOPN has been identified in about 70% of *C*. *jejuni* CPSs[6]. Moreover, genes encoding MeOPN transferases also have been identified, and appear to modulate the Penner serotype within the HS23/36 complex (e.g., *C*. *jejuni* strain 81–176), likely because of phase variation in expression[4, 10]. A study suggests that the capsular heptose modification pathway contributes to bacterial resistance against gastrointestinal host defenses and supports bacterial persistence via its role in serum resistance and invasion of intestinal cells[11].

There are reports indicating that *C*. *jejuni* strains associated with GBS belong to specific Penner serotypes[12]. In 2007, 36 cases of GBS occurred in Shuangyang, a township in Changchun, Jilin Province, China and *C*. *jejuni* of HS41 was collected[13, 14]; in 2011, 26 cases of GBS were identified in San Luis Río Colorado, Sonora, Mexico and Yuma County, Arizona, USA, and *C*. *jejuni* of HS4 was isolated[15]. Studies based on Penner serotyping of GBS in Japan, Bangladesh and South Africa showed that HS19, HS23/36c and HS41 were the major capsular serotypes, respectively[16–18]. The serotypes of HS4, HS2, and HS1 identified in sporadic cases were reported as the most common in the world [7]. Serotype HS2, HS19 and HS41 are the entire serotypes which were recognized as the ones highly associated with GBS cases in China as well as in other regions.

In a primate model, it was demonstrated that a CPS conjugate vaccine can protect against *C*. *jejuni* diarrhea[19]. But the cost and complexity of producing the required antisera have limited usefulness of Penner serotyping scheme which is based on CPS loci type. In this study, we analyzed the genetic diversity of the CPS locus in *C*. *jejuni* isolates from China and developed a multi-PCR assay to identify specific serotypes of *C*. *jejuni*.

## Materials and Methods

### Ethics statement

There were no human tissue or blood samples used in this study. All the bacteria isolates used in this study were previously isolated from the stool specimens. The isolates from the patients were isolated from the stool specimens by the clinical laboratories and the isolates from the chicken fecal matter were collected from the inspection labs from local CDCs which were involved in the project for Campylobacter surveillance in China. Because the collection of stool specimens is noninvasive, verbal consent was acquired from all patients. This research project was approved by the ethics committee of the China CDC and the academic committee in the National Institute for Communicable Disease Control and Prevention. All the related documents were recorded at the China CDC (ICDC-2014011, ICDC-2014012).

### Bacterial strains and genomic DNA purification

The isolates were previously collected from stool samples of GBS patients (HB-CJGB-LL, BJ-CJGB96G25, HB-CJGB-XWM, ICDCCJ07001), stool samples of diarrheal patients (BJ-CJD63, BJ-CJD70, BJ-CJD39, BJ-CJD120), and chicken feces (JL-CJHLIU1-1) in China. The Penner serotypes of these isolates were determined using a commercial 25 Penner heat-stable antisera set (*Campylobacter* Antisera Seiken Set; Denka Seiken, Japan). Strains were selected for sequence analysis based on their different serotypes, including three serotypes related to GBS (HS2, HS19, HS41)[12] and five serotypes whose CPS sequences have not been studied previously (HS9, HS12, HS21, HS31, HS37). Information on CPS sequences of other serotypes was obtained from the GenBank database (http://www.ncbi.nlm.nih.gov/). All the CPS sequences were compared for the existence of conserved genes (e.g., heptose, fucose, and MeOPN biosynthesis genes). Information on the studied isolates is summarized in Table 1.

**Table 1:** Summary of sequenced *C*. *jejuni* CPS loci.

Strain	Serotype	Size(bp)	Accession No.	GC(%)	No. of genes	No. of MeOPN transferases	genes for synthesis of Heptose	genes for synthesis of Deoxyheptose	No. of sugar transferases
BJ-CJD120	HS1/44	11756	LISM00000000	27.2	12	1	No	No	3
NCTC11168	HS2	34180	NC_002163	26.5	28	2	Yes	No	8
GC8421	HS3	26371	ABGQ01000005	27.3	22	1	Yes	Yes	10
GC8486	HS4	23423	AASY01000000	28	19	2	Yes	Yes	4
81116	HS6	26729	NC_009839	27.6	22	0	No	No	8
BJ-CJD63	HS9	27156	LISK00000000	26.3	24	0	No	No	6
ATCC 43444	HS8/17	22063	HQ343270	27.1	18	0	Yes	Yes	5
ATCC 43438	HS10	27307	HQ343271	27.1	25	1	Yes	Yes	4
BJ-CJD70	HS12	23847	LISL00000000	26.9	20	0	Yes	Yes	7
ATCC 43442	HS15	23868	HQ343272	28.3	22	1	Yes	Yes	5
BJ-CJGB96G25	HS19	16724	ASXL00000000	26.0	13	1	No	No	4
JL-CJHLIU1-1	HS21	24407	LISQ00000000	27.6	19	1	Yes	Yes	3
81–176	HS23/36	24625	NC_008787	27.1	23	1	Yes	Yes	7
BJ-CJD39	HS31	29413	LISI00000000	27.2	29	1	Yes	Yes	2
HB-CJGB-LL	HS37	35109	ATBJ00000000	25.7	31	2	No	No	6
ICDCCJ07001	HS41	34196	CP002029	27.0	31	0	Yes	Yes	5
ATCC 43461	HS42	23268	HQ343274	26.9	21	0	Yes	Yes	7
HB-CJGB-XWM	HS53	18303	ATBN00000000	26.3	15	0	Yes	Yes	7

Isolates were grown on Karmali selective medium (Oxoid CM0935B), supplemented with 5% sheep blood at 42°C in microaerobic conditions (5% O_2_, 10% CO_2_, and 85% N_2_) for 48 h. Total DNA was extracted from each isolate using the QIAamp DNA mini kit (Qiagen, Hilden, Germany) according to the manufacturer’s protocol.

### Genome sequencing and CPS locus identification

The isolates in this study were sequenced using an Illumina HiSeq 2000 sequencing platform by Beijing Genomics Institution with a depth of 450× coverage. The number of reads are between 11700000 and 12000000. Low quality reads were removed if the quality scores of ≥3 consecutive bases were ≤Q30. The Illumina reads were then assembled into contigs and scaffolds by using SOAP*denovo* v2.04 (http://soap.genomics.org.cn/soapdenovo.html). PCR amplification and Sanger sequencing were used for filling the gaps in the region of CPS sequences. Genes were predicted using Glimmer[20], and functional annotation was performed by comparison with COG, KEGG, and Nr databases using BLAST software[21]. The assembled genomes have been submitted to the GenBank database with the accession numbers listed in Table 1. The sequences of other isolates in Table 1 were downloaded from GenBank (http://www.ncbi.nlm.nih.gov/).

Following Karlyshev[4], the conserved CPS biosynthesis genes *kpsF* and *kpsC* were considered as the first and last genes of the CPS gene cluster, respectively. *KpsF* and *kpsC* were found in the whole genome sequence and the sequence between these two genes was assigned as the DNA sequence of the CPS locus. A database was created containing all the 18 sequenced serotype CPS gene clusters.

### Orthology assignment

OrthoMCL v2.0.9[22] was used to cluster all CPS genes into orthologous groups including 13 serotypes (HS1, HS2, HS23/36, HS19, HS41, HS42, HS3, HS6, HS8/17, HS10, HS15, HS4/13/64, HS53) from previous studies and five serotypes (HS9, HS12, HS21, HS31, HS37) sequenced in this study. Firstly, the protein sequences were compared with each other using the software BLASTp. A normalized similarity matrix was built based on the e-values (cut-off of 1e-5) using a Markov Cluster algorithm, in which the proteins were assigned into groups of orthologs and paralogs[23]. A database was created containing orthologs and paralogs, respectively. Then, we compared the sequences in which the “ortho groups” of CPS loci are similar using BLAST software for specific serotypes (HS9, HS12, HS21, HS31, HS37).

### DNA BLAST and PCR development

We selected paralogs from the orthology assignment results as unique genes in each serotype. Then, these genes were subjected to a simple BLAST[24] search against the GenBank database (http://blast.ncbi.nlm.nih.gov/Blast.cgi) to eliminate genes that mapped onto other *Campylobacter* spp. Primers were designed based on the sequences of the remaining unique genes from each serotype. For serotype HS37, for which no unique genes were detected using this method, we found a region that is unique based on which one primer was designed. For the non-unique gene of the serotype HS21, we found a region that mapped onto a strain of *C*. *lari* only, so we designed one primer in this region and the other primer outside this region. Primers were designed using Primer Premier 5.0[25] software with the following parameters: length between 18 and 25 residues; 40 to 60% GC content; melting temperature ranging from 55 to 65°C; verification of the absence of dimerization or hairpin formation. We used Oligo 7[26] software to check primer dimerization based on Gibbs free energy (ΔG) with a stringent cutoff value of -6.5 kcal/mol to define a dimer. The multi-PCR primer sets were designed into two mixes, Set A and Set B, and the PCR products differ from each other by at least 90 bp in the same mix.

The Multi-PCR system was optimized using 12.5 μL of 2×Easy*Taq* PCR SuperMix (Transgen, Beijing, China), 6.5 μL (Set A) or 8.5 μL (Set B) of ddH2O, 0.5 μL of each primer (1 OD at 260nm), and 1 μL of DNA template in a total volume of 25 μL run with the following parameters: 94°C for 4 min, followed by 30 cycles of 94°C for 1 min, 55°C for 1 min and 72°C for 45 s, followed by a final extension at 72°C for 8 min.

### Validation of the multiple-PCR assay

Two hundred and twenty-seven *C*. *jejuni* isolates were obtained from clinical diarrheal patients and chickens. The serotypes were determined in the lab then used to verify the PCR system. DNA was prepared by the whole-cell procedure. Half of one loop of culture was removed and emulsified in 100 μL of distilled sterile water in a 0.5-mL microcentrifuge tube and heated to 100°C for 10 min. Templates were used immediately for PCR.

## Results

### Genetic diversity of the CPS

**Fig 1:**
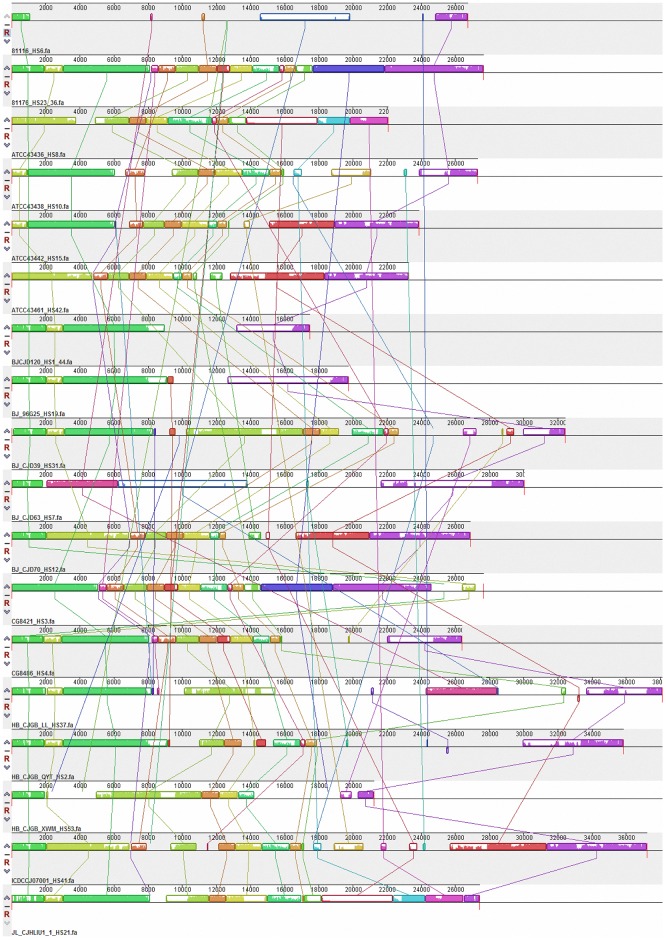
Map of 18 CPS loci. The same color module in different strains means that the sequences in this region are similar. Blank regions indicate a unique region in a sequence. The name of the sequences is made up of the strain ID and serotype. The comparison was made using Mauve 2.4.0 software.

An overview of 18 CPS loci is shown in Fig 1. High similarity of sequence is indicated by the same color and unique regions are in white. Considering the five specific serotypes we newly sequenced, HS37 has more similar regions with HS9 and HS31 than with other serotypes; HS12 is related to HS42; and HS21 has high similarities with parts of HS15 and HS8/17.

**Fig 2:**
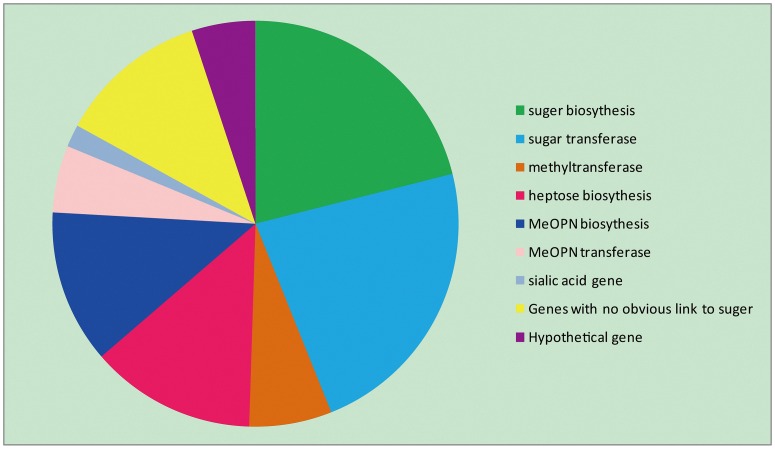
Distribution of CPS gene functions. Different colors represent different gene functions. The proportion of a color indicates the percentage of genes with the corresponding function.

**Fig 3:**
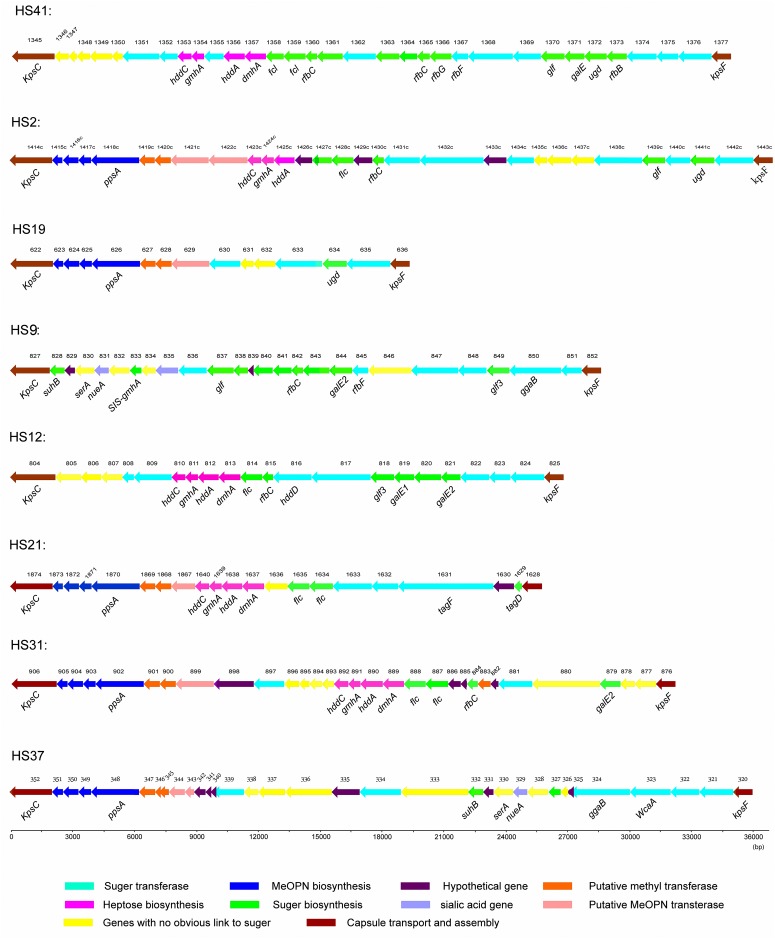
Schematic diagram of variable CPS loci from representative Penner serotypes. Gene names were attributed if the predicted protein showed >80% sequence identity with other known *C*. *jejuni* proteins. Genes are color coded as shown in the key to the Fig on the basis of best homology to any predicted protein in databases by BLAST analyses.

**Fig 4:**
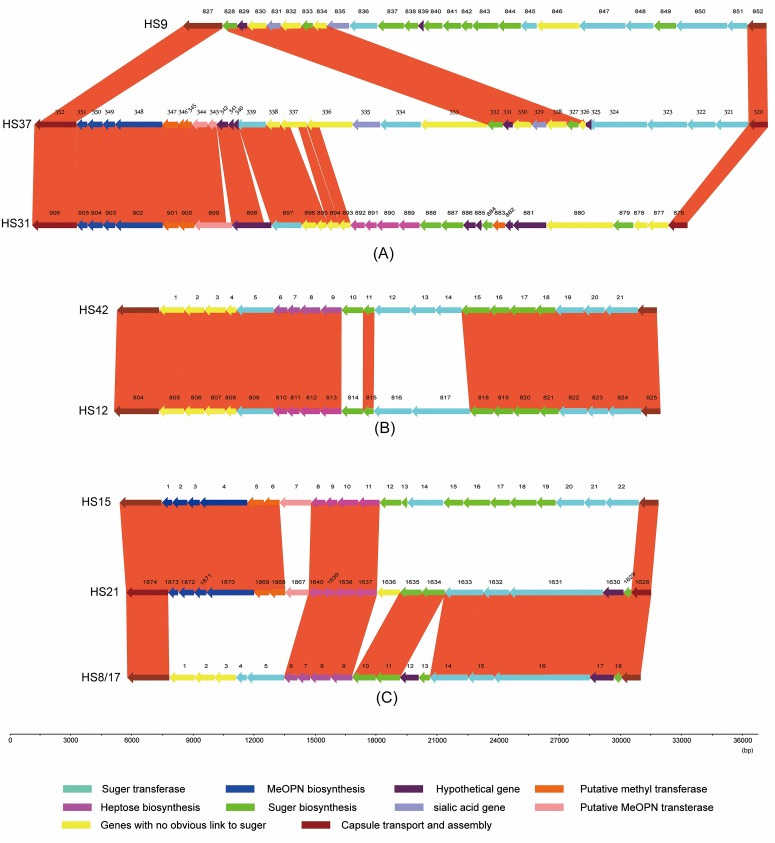
Comparison of sequences of CPS loci of *C*. *jejuni* strains. (A) Comparison of the sequences of the CPS loci of *C*. *jejuni* HS9 (strain BJ-CJD63), HS37 (strain HB-CJGB-LL) and HS31 (strain BJ-CJD39); (B) comparison of the CPS loci of *C*. *jejuni* HS12 (strain BJ-CJD70) and a strain of HS42 (strain ATCC 43461); (C) comparison of the CPS loci of *C*. *jejuni* HS21 (strain JL-CJHLIU1-1), the HS15 type strain (strain ATCC 43442), and an HS8/17 strain (strain 43444). Red bars represent identical regions between loci.

There are a total of 394 open reading frames (ORFs) present in the CPS loci of 18 serotyped strains. The main functions of these ORFs are shown in Fig 2. Most of these genes are related to sugar biochemistry (including sugar biosynthesis genes and sugar transferase genes) and MeOPN (including MeOPN biosynthesis genes, MeOPN transferase genes and methyltransferase genes), accounting for 43.9% and 24.1% of the total genes, respectively. We also detected seven genes (1.5%) related to sialic acid functions, distributed in HS6 (3/7), HS9 (2/7), and HS37 (2/7). The rest of the genes are genes with no obvious link to sugar chemistry or are hypothetical proteins. Schematics of the CPS loci from representative Penner serotypes (three GBS marker serotypes and the five serotypes we sequenced), which were variable, are shown in Fig 3. Combining the results from Figs 1 and 3, the specific serotypes we sequenced were compared by BLAST software (Fig 4).

### Diversity of CPS gene clusters

**Fig 5:**
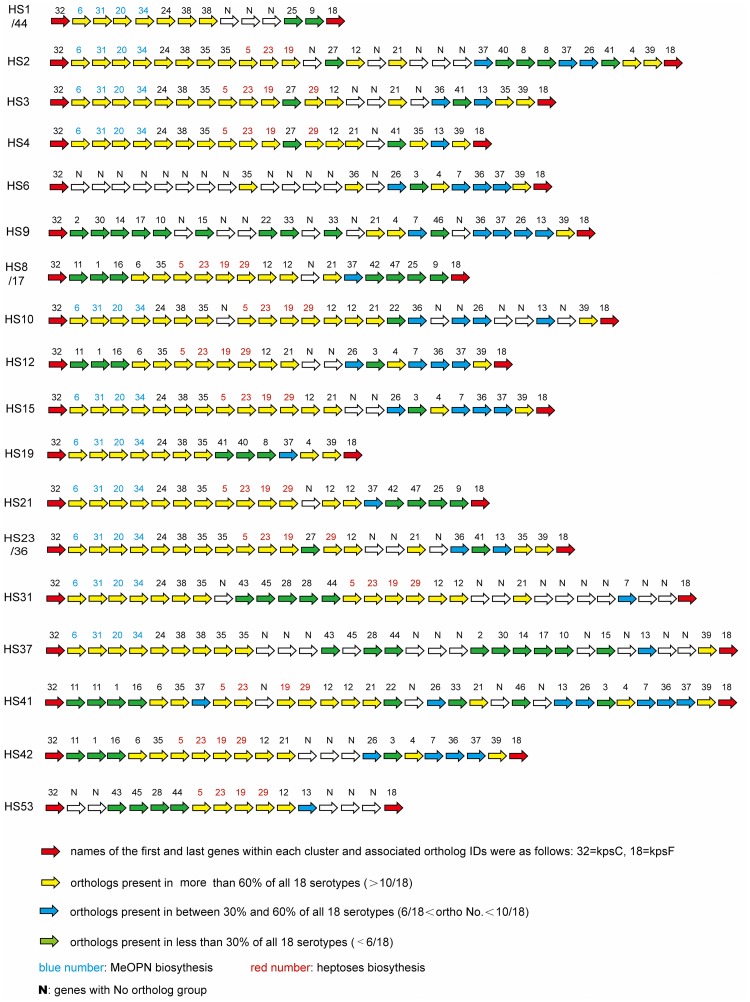
Ortholog content in the CPS gene cluster. Arrows represent orthologs, and numbers are ortholog ID numbers (see Sheets A-C in S1 Dataset for associated annotations). Red arrows: orthologs belonging to the core genome (i.e., orthologs seen in all strains), with all remaining arrows representing orthologs belonging to the dispensable genome. Orthologs are color coded by percentage presence in all 18 serotypes with the separation 30% and 60%; genes with no color are paralogs.

In total, 47 orthology groups were detected in the 18 serotypes (Fig 5). Actual ORFs and associated orthology groups for each stain sequenced are shown in Sheet A of S1 Dataset. The number of serotypes represented in each ortholog group and the function of each ortholog group are shown in Sheet B of S1 Dataset. Seventy-seven paralogs, which means that the genes were not put into “ortholog groups”, were also detected. The paralogs can be thought of as a marker for one serotype. The function of each paralog is shown in Sheet C of S1 Dataset.

### Multiple-PCR validation

**Fig 6:**
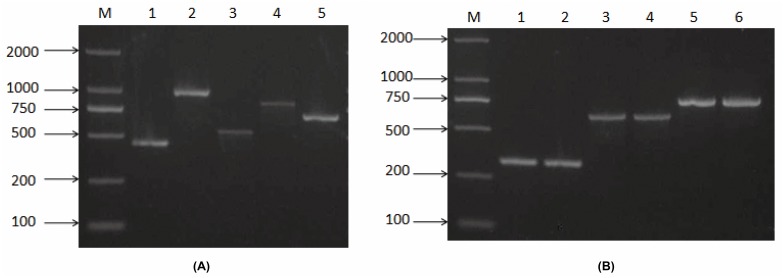
**(A)Typical PCR products obtained with primer mix Set A.** Lane M: DL2000 DNA marker; Lanes 1, 2: HS41; Lanes 3, 4: HS19; Lanes 5, 6: HS2. **(B) Typical PCR products obtained with primer mix Set B.** Lane M: DL2000 DNA marker; Lane 1: HS9; Lane 2: HS21; Lane 3: HS37; Lane 4: HS12; Lane 5: HS31.

The primers designed in this study are listed in Table 2. The primer sets were grouped into two mixes on the basis of GBS-related group serotypes and the five serotypes newly sequenced in this study (Table 2). Thus, the Set A mix contained primers that distinguish HS2, HS19, and HS41. The Set B mix contained primers that distinguish HS9, HS12, HS21, HS31 and HS37. Following amplification, Set A and Set B mix PCRs were run separately on 1.5% agarose gels in parallel with DL2000 DNA marker (TaKaRa, Dalian, China) to decipher the capsule type. Amplicon sizes in Set A ranged from 328 bp for HS41 to 780 bp for HS2 (Fig 6A). Amplicon sizes in Set B ranged from 460 bp for HS9 to 989 bp for HS21 (Fig 6B). Expected sizes are listed in Table 2.

**Table 2:** Information of multiple-PCR primers.

Mix	Product size (bp)	ORF in which primer was designed	Penner type recognized	Forward sequence	Reverse sequence
Set A					
ICDCCJ_HS2	780	cj1431c	HS2	CCCCGCCAGTAGTTAAGGT	AATGAGGCTACGATTCCGC
ICDCCJ_HS19	649	BJ-CJGB96G25-GL000630	HS19	GAATGCGTTATGAGCAACAGGAT	GATCATCATCAAGCCTTTGC
ICDCCJ_HS41	328	ICDCCJ07001_1363	HS41	AGATGTATGGAAGGTATGTGGTC	TAAATGGGGTGCTCGTGAA
Set B					
ICDCCJ_HS9	460	BJD63GL000835	HS9	TCCCCATCACCATAGGCTA	ACAATCCGTCTTTCGCAAT
ICDCCJ_HS12	835	BJD70GL000816	HS12	GCAGCCAAACTATAACGCATTA	AAAGTGGGCGGCAAATAGTAGA
ICDCCJ_HS21	989	1-1GL001635;1-1GL001636	HS21	TACTCCACGATACCGCAGG	TTATGGTTCTGCTTGGGCT
ICDCCJ_HS31	707	BJD39GL000882	HS31	AGGTAAATCAGGTCTTGTGGG	TAAAAGAGTGGGAGGAAGCA
ICDCCJ_HS37	550	HB-CJGB-LLGL000322	HS37	GATAAGGAAAACGGCGGTCT	CAAAATGGCAATCTTCAGCA

To validate the multiple-PCR, Set A and Set B mixes were tested on 227 previously serotyped strains. Seventy-one of these strains were typed as one of the CPS types included in the multiple-PCR, and the other strains were serotypes that were not included in the multiple-PCR. The results are summarized in Table 3. All serotypes detected were true positives and sensitivity/specificity are 100%.

**Table 3:** Validation of CPS multiple-PCR.

	Total	True positive	True negative	Accuracy
HS2	25	25	202	100%
HS19	11	11	216	100%
HS41	5	5	222	100%
HS9	13	13	214	100%
HS12	4	4	223	100%
HS21	2	2	225	100%
HS31	6	6	221	100%
HS37	5	5	222	100%

## Discussion

Previous study has demonstrated evidence of the exchange of capsular genes by horizontal gene transfer (HGT)[4]. It was considered that the dispensable genome would turn over rapidly due to the frequent HGT[27]. Intraspecies recombination among CPS loci of *C*. *jejuni* predominated within dispensable components of the genome[28]. In this study, genes were regarded as dispensable for each serotype if they were not shared among all CPS loci. Genes contained by all CPS loci were considered as core genes. With the exception of genes of *kpsC* and *kpsF*, all CPS genes belonged to the dispensable genome.

Bacteria shape their gene content diversity by gene gain and loss[29]. Fig 1 shows that many highly similar genes/gene clusters (identically colored regions) and unique genes/gene clusters (blank regions) occur in different CPS loci. There were 394 ORFs within CPS loci and 317 (80.5%) of these form orthologs (Fig 5). Paralogs, also shown in Fig 5, account for 19.5% of the genes (77/394). HS6, HS31 and HS37 contain more paralogs (13, 9 and 11, respectively) than the other serotypes (<7). Two probable explanations for these observations are frequent HGT among CPS genes, and gene gain/loss events that are increasing the gene content diversity of each CPS locus.

The results in Fig 5 also indicate that 61.1% (11/18) of the CPS loci contain orthologs of groups 6, 31, 20 and 34 (the orthology group numbers are shown in supplement 1, similarly hereinafter) which are annotated with the function of MeOPN biosynthesis genes. Orthologs of group 35 are usually adjacent to the MeOPN gene cluster and encode MeOPN transferases[9]. The conserved N-terminal region were likely to encode the enzyme to recognize MeOPN and the variable C-terminal region was likely to reflect the different sugar residues to which the MeOPN is attached[7]. Other highly conserved orthologs in various CPS loci are the genes encoding enzymes for biosynthesis of heptose (*hddC*, *gmhA2 and hddA*) and deoxyheptose (*dmhA*). We found that 72.2% (13/18) of the CPS loci contain *hddC*, *gmhA2* and *hddA*, the orthology group numbers of which are 5, 23 and 19, respectively. Orthology group 29, which is annotated with the gene *dmhA*, is present in 66.7% of the strains (12/18).

The HS9 CPS locus contains 24 genes, including arabinose, pyranose and galactopyranose biosynthesis genes. However, HS9 has no conserved gene cluster with genes for MeOPN or biosynthesis of heptose and deoxyheptose. HS9 contains six paralogs. The HS31 CPS locus contains 29 genes, including MeOPN, heptose, deoxyheptose, pyranose and fucose biosynthesis genes. Unlike other CPS loci, there is a gene (see Sheet C of S1 Dataset) encoding a methyl transferase which is not adjacent to the MeOPN gene cluster. The HS37 CPS locus contains 31 genes, including MeOPN and pyranose biosynthesis genes, but HS37 has no heptose or deoxyheptose biosynthesis genes.

The HS9, HS31 and HS37 CPS loci were compared using BLAST software (Fig 4A). The HS37 CPS locus appeared to be a mosaic between HS9 and HS31. Seven genes in the middle of the HS37 CPS locus show a high degree of similarity with the first seven genes in the HS9 locus, including a sialic acid-related gene *nueA* (N-acylneuraminate cytidylyltransferase) and a sugar biosynthesis gene *SIS-gmhA* (D-glycero-D-mannoheptose 7-phosphate isomerase). The ORFs (see Sheet A of S1 Dataset) of HS37 from HB-CJGB-LLGL000352 to HB-CJGB-LLGL000336 show a high degree of, or partial similarity with, those in the HS31 CPS locus from BJD39GL000906 to BJD39GL000893. In this region, the ORFs HB-CJGB-LLGL000333 and HB-CJGB-LLGL000334 in HS37 mapped partly onto ORF BJD39GL000899 in HS31; the three ORFs are all putative methyl transferases. The ORFs BJD39GL000893 and BJD39GL000894 in HS31 mapped partly onto ORF HB-CJGB-LLGL000337 in HS37; ORF BJD39GL000893 in HS31 mapped partly onto ORF HB-CJGB-LLGL000336 in HS37.

The HS12 CPS locus is related to the HS42 locus (Fig 4B), including putative genes for synthesis of arabinose, heptose and deoxyheptose. The major differences are that two putative sugar transferases (glycosyl transferase and O-fucosyltransferase) that are absent from HS42 are present in HS12, and that HS12 lacks four genes found in HS42. The ORFs HS42.10 and BJD70GL000814 are in the same ortholog group but they did not map to each other. These two genes may be isoenzymes of GDP-fucose synthetase. The capsule locus of HS12, like that of HS42, lacks genes for MeOPN synthesis.

The HS21 CPS locus appeared to be a mosaic between HS15 and the HS8/17 complex (Fig 4C). The first six genes of HS21 mapped with a corresponding region of HS15. The last six genes of HS21 are highly similar to those genes in HS8/17. The three CPS loci are conserved with each other in the region of *hddC* to *dmhA*. The HS21 CPS locus contains two fucose biosynthesis genes, as do HS8/17. ORFs 1-1GL001867 and 1-1GL001636 are two unique genes in HS21, encoding methyltransferase and pyridoxamine 5-phosphate oxidase enzymes, respectively.

Studies on risk factors for GBS showed that sialylation of lipooligosaccharides (LOS) of *C*. *jejuni* is considered to be the most important virulence factor[30, 31]. The molecular mimicry between sialylated *C*. *jejuni* LOS and ganglioside structures of peripheral nerves causes the autoimmune neuropathy[30]. In *C*. *jejuni*, genes for sialylation are located in the biosynthesis locus of the LOS cluster[32]. However, sialylation is not the only factor in *C*. *jejuni* involved in the pathogenesis of GBS. It was reported that up to 63% of *C*. *jejuni* strains that caused only uncomplicated enteritis carried the genes for sialylation biosynthesis[31]. The capsular type was related to the LOS class in order to determine whether particular LOS class/capsular type combinations were more prevalent amongst GBS-associated strains than among enteritis-associates strains[12]. CPS are considered to be crucial *C*. *jejuni* virulence factors associated with GBS and capsular types HS1/44c, HS2, HS4c, HS19, HS23/36c and HS41 are markers for GBS[12]. In this study, we also find some genes related to sialic acid in CPS gene clusters. Each of the CPS loci of HS6, HS9 and HS37 contain one sialic acid biosynthesis gene, *nueA*, encoding N-acylneuraminate synthetase. Another sialic acid biosynthesis gene, *synX* (N-acetylglucosamine-6-phosphate 2-epimerase), was found in HS6. There are two genes encoding α-2,3-sialyltransferase present in each HS9 and HS37. Usually, sialic acid-related genes are extensively detected in the LOS of *C*. *jejuni*[31, 32]. It is possible that HGT between the LOS and CPS leads to sialic acid genes incorporating into the CPS cluster.

The traditional serotype identification method depends on cross-reacting serotype complexes, which is time and labor intensive requiring repeated tests[33]. Previous study has designed multiple-PCR to identify 14 serotypes, separated into two mixes, alpha and beta[6], in lieu of classical serotyping. In this study, we develop multiple-PCR to identify more serotypes of *C*. *jejuni* (including HS9, HS12, HS21, HS31 and HS37) and analyze the features and relationships of the DNA sequences of their CPS loci and also of three GBS-related serotypes (HS2, HS19 and HS41).

The strategy was to detect paralogs or to find a unique sequence region in one CPS cluster sequence as the unique marker of a serotype and then design specific primers based on that. Each primer set was tested on the strain from which it was designed and 13 serotypes identified in previous studies (see Sheet A of S2 Dataset) to confirm specificity for these serotypes. We do not have any other serotype isolates, so we cannot test further serotypes. The primer sets were considered to be valid if their PCR products were the expected size of the target DNA for each specific serotype or the related complexes. If the product were negative, then the strains belonged to the other serotype. Data are shown in Sheet A of S2 Dataset showing that there are no cross reaction. The multiple-PCR method had specificity, accuracy and sensitivity of 100% for the 71 strains that serotyped as one of the eight serotypes analyzed in this work and covered in the multiple-PCR (Table 3), showing that the method is accurate and effective.

## Conclusions

In this study, the CPS loci from five (HS9, HS12, HS21, HS31 and HS37) different *C*. *jejuni* Penner serotypes were sequenced. In order to distinguish these serotypes efficiently and accurately, we designed a multiple-PCR method. Including data for another 13 serotypes/serotype complexes from previous studies, analysis of 18 CPS loci shows extensive gene gain/loss events among them, with the lateral transfer of genes. The CPS locus of HS21 is a mosaic of HS15 and HS8/17, and that of HS37 is a mosaic of HS9 and HS31. Analysis of gene homology shows that the MeOPN biosynthesis genes and heptose biosynthesis genes are consistently highly conserved genes, and HS9, HS31 and HS37 have more paralogs revealing more variation in evolution. The finding of sialic acid-related genes in CPS loci raises the possibility of HGT between LOS and CPS clusters and highlights the potential for GBS in strains of HS6, HS9, HS31 and HS37.

Studies on the features of CPS types will provide useful and significant data to promote the development of a CPS conjugate vaccine against *C*. *jejuni*-induced diarrhea and other Campylobacteriosis. We also designed a multiple-PCR for the GBS-related serotypes HS2, HS19 and HS41. We expect to detect these serotypes more simply in future emergencies or outbreaks in order to control the potential occurrence of GBS in a timely manner.

## Supporting Information

S1 Dataset Summary of ORFs of the 18 CPS loci. Sheet A contains all the ORFs of the 18 CPS loci with their orthology groups. Sheets B and C contain the functions of orthologs and paralogs, respectively.
(XLSX)

S2 Dataset Verification of each multiple-PCR primer pairs. Summary of PCR amplification from multiple-PCR primer pairs developed in this study. The strains with asterisks are unpublished strains (Zhang M. et al).
(XLSX)
